# Impact of Adolescent Intermittent Ethanol Exposure on Social Investigation, Social Preference, and Neuronal Activation in cFos-LacZ Male and Female Rats

**DOI:** 10.3389/fphar.2022.841657

**Published:** 2022-03-23

**Authors:** Trevor T. Towner, Devon T. Applegate, Elena I. Varlinskaya, David F. Werner

**Affiliations:** Neurobiology of Adolescent Drinking in Adulthood Consortium, Center for Development and Behavioral Neuroscience, Department of Psychology, Binghamton University, Binghamton, NY, United States

**Keywords:** adolescence, ethanol exposure, social behavior, sex differences, cfos, βgalactosidase

## Abstract

Adolescence is a sensitive developmental period during which alcohol use is often initiated and consumed in high quantities, often at binge or even high-intensity drinking levels. Our lab has repeatedly found that adolescent intermittent ethanol (AIE) exposure in rats results in long-lasting social impairments, specifically in males, however our knowledge of the neuronal underpinnings to this sex-specific effect of AIE is limited. The present study was designed to test whether social anxiety-like alterations in AIE-exposed males would be accompanied by alterations of neuronal activation across brain regions associated with social behavior, with AIE females demonstrating no social impairments and alterations in neuronal activation. Adolescent male and female cFos-LacZ transgenic rats on a Sprague-Dawley background were exposed to ethanol (4 g/kg, 25% v/v) or water *via* intragastric gavage every other day during postnatal days (P) 25–45 for a total of 11 exposures (*n* = 13 per group). Social behavior of adult rats was assessed on P70 using a modified social interaction test, and neuronal activation in brain regions implicated in social responding was assessed *via* β-galactosidase (β-gal) expression. We found that AIE exposure in males resulted in a significantly lower social preference coefficient relative to water-exposed controls, with no effect evident in females. Exposure-specific relationships between social behavior and neuronal activation were identified, with AIE eliminating correlations found in water controls related to social interaction, and eliciting negative correlations mainly in limbic regions in a sex-specific manner. AIE exposure in the absence of social testing was also found to differentially affect neural activity in the orbitofrontal cortex and central amygdala in males and females. These data suggest that AIE produces sex-specific social impairments that are potentially driven by differential neuronal activation states in regions important for social behavior, including the medial prefrontal and orbitofrontal cortices, nucleus accumbens, lateral septum, and central amygdala. Future studies should be focused on identification of specific neuronal phenotypes activated by interaction with a social partner in AIE-exposed subjects and their control counterparts.

## Introduction

Adolescence is a sensitive developmental period during which the brain undergoes substantial maturation (Larsen and Luna, 2018) and is especially vulnerable to different insults including alcohol. Alcohol use is often initiated in adolescence (Morean et al., 2018), with over 2 million adolescents in the United States between 12 and 17 years of age initiating alcohol in 2019 (Substance Abuse and Mental Health Services -SAMHSA, 2020). During late adolescence alcohol use becomes heavier, often in the forms of binge (SAMHSA, 2020) and high intensity drinking (Patrick and Terry-McElrath, 2019). These more risky drinking patterns are associated with short-term cognitive deficits (Jones et al., 2018), however less is known about the long-term alterations as a result of adolescent alcohol exposure.

Preclinical rodent models have identified several long-lasting behavioral impairments associated with adolescent alcohol (ethanol) exposure (Crews et al., 2019), including, but not limited to, increases in anxiety-like behavior (Kyzar et al., 2019; Sakharkar et al., 2019; Varlinskaya et al., 2020), reduced learning, memory, and cognitive flexibility (Gass et al., 2014; Fernandez et al., 2017; Fernandez and Savage, 2017; Rico-Barrio et al., 2019; Macht et al., 2020; Varlinskaya et al., 2020), and also increased risk-taking (Nasrallah et al., 2011; Clark et al., 2012; Kruse et al., 2017). These behavioral impairments are also associated with numerous neurobiological changes such as decreased acetylcholine markers (Vetreno and Crews, 2018; Vetreno et al., 2020; Kipp et al., 2021), increased neural inflammation (Vetreno and Crews, 2012; Vetreno et al., 2017; Vetreno and Crews, 2018; Macht et al., 2020; Vetreno et al., 2020), and dysregulated epigenetic regulators (Kokare et al., 2017; Kyzar et al., 2017; Kyzar et al., 2019).

Our lab has found that adolescent intermittent ethanol (AIE) induces alterations of social behavior in adulthood (Varlinskaya et al., 2014; Varlinskaya et al., 2017; Dannenhoffer et al., 2018; Varlinskaya et al., 2020). These AIE-induced social impairments are sex-specific and evident only in males. These social deficits are reversible through systemic administration of either an oxytocin (OXT) agonist or vasopressin receptor 1b (AVPR1b) antagonist (Dannenhoffer et al., 2018). Furthermore, AIE leads to decreased hypothalamic OXT receptor and increased AVPR1b protein membrane surface expression (Dannenhoffer et al., 2018), specifically in males, findings likely contributing to the social deficits evident in these animals. However, our understanding of the neural mechanisms underlying AIE-induced social deficits is still limited.

Social behavior is regulated by various brain regions that together form the social brain network (Newman, 1999). The social brain network was originally outlined as being composed of the extended amygdala, lateral septum (LS), hypothalamus (HYPO), preoptic area, and midbrain (Newman, 1999). Although evidence supports the involvement of these regions in the regulation of social behavior, more recent evidence also indicates regions outside of this network that contribute to social behavior (Yizhar, 2012; Allsop et al., 2014; Vanderschuren et al., 2016; Ko, 2017). For example, social interaction (approach and contact with a social partner) in males elicits greater firing of neurons in the prelimbic (PrL) cortex (Minami et al., 2017), with similar findings of increased firing of projections from the orbitofrontal cortex (OFC) to basolateral amygdala (BLA) during social approach in males (Li et al., 2021). Furthermore, chemogenetic activation of PrL to BLA circuitry reduced social preference in females (Huang et al., 2020) whereas optogenetic activation of the PrL to nucleus accumbens (NAc) pathway in both sexes leads to decreased social investigation (Murugan et al., 2017). Lesioning the PrL, thus disrupting all afferent and efferent pathways of the PrL, leads to increased social interaction among males (Shah and Treit, 2003) although baclofen and muscimol mediated inhibition of the PFC, including the PrL, infralimbic (IL), and OFC, reduced social play behavior in adolescent males (Van Kerkhof et al., 2013). Taken together, recent evidence suggests that select PFC neural ensembles and their connections to limbic and amygdalar regions play a large role in the regulation of social behaviors.

Given our previous sex-specific findings of AIE-associated alteration of the OXT and AVP systems, it is also possible that regions with known sex differences in these neuropeptide systems are differentially involved in modulation of social interaction in AIE-exposed males relative to controls as well as differences between control conditions in each sex. For example, the LS receives greater AVP innervation from the bed nucleus of the stria terminalis (BNST) (De Vries et al., 1981; De Vries and Buijs, 1983) and HYPO (Qiao et al., 2014) in male than in female rodents. Sex differences in the expression of OXT and AVP receptors are also evident across multiple brain regions, including the PFC, subregions of the BNST, NAc, medial amygdala (MeA), and ventromedial HYPO (Dumais et al., 2013; Dumais and Veenema, 2016). In addition, AVP synthesis in regions such as the BNST has been shown to be different between sexes (Van Leeuwen, Caffe, and De Vries, 1985). However, it is still unknown whether neuronal activation within regions that show sex differences in the OXT and AVP systems in response to a social stimulus differs as a function of adolescent ethanol exposure and sex, and whether these brain regions contribute to AIE-induced social alterations in males.

**FIGURE 1:**
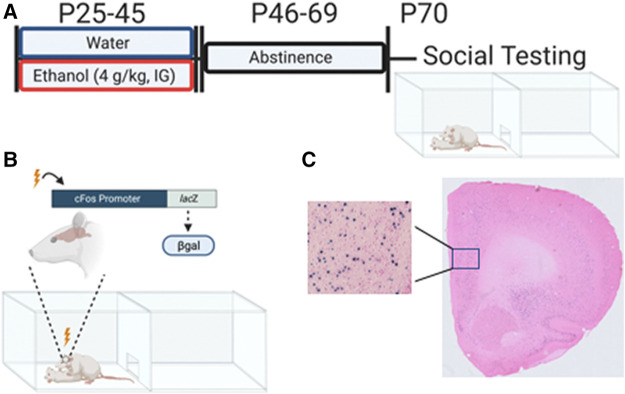
Adolescent intermittent ethanol exposure and β-gal expression associated with social interaction in adulthood. **(A)** Experimental timeline of adolescent intermittent ethanol exposure and social interaction testing in adulthood. **(B)** cFos-LacZ transgenic model allows for measuring β-galactosidase (β-gal) expression as a proxy for cFos. **(C)** Representative X-gal enzymatic labeling of active neurons containing (β-gal) following interaction with an unfamiliar social partner. Figure was created using BioRender.com

We hypothesized that ethanol exposure during adolescence may affect the brain in a sex-specific manner, with brain regions implicated in social behavior regulation, especially those that show sex differences, being sensitive to AIE in males. The current study was designed to assess neuronal activation induced by a social stimulus in “social” brain regions of adult male and female cFos-LacZ rats exposed to AIE. In these transgenic rats, expression of β-galactosidase (β-gal) is under the control of a *cfos* promoter (Figure 1B), and cFos and β-gal proteins are co-expressed in neurons strongly activated by a certain stimulus (Cruz et al., 2015). Therefore, β-gal expression can be used as a proxy for cFos. While cFos expression requires immuno-histochemical techniques, β-gal is detected by X-gal staining–a rapid and highly reproducible histochemical assay. In this study, β-gal expression was assessed in the following regions of interest (ROIs): prelimbic cortex (PrL), infralimbic cortex (IL), ventral and lateral orbitofrontal cortex (vOFC, lOFC), nucleus accumbens core (NAcC), nucleus accumbens shell (NAcSh), lateral septum (LS), dorsal bed nucleus of the stria terminalis (dBNST), anterior hypothalamus (AH), central amygdala (CeA), and basolateral amygdala (BLA).

## General Methods

### Subjects

Male and female adolescent cFos-LacZ transgenic rats on a Sprague-Dawley background bred and reared in our colony at Binghamton University were used. cFos-LacZ rats have an *E. coli* derived LacZ gene with expression driven by a cFos promoter allowing for simultaneous transcription of cFos and LacZ. This pairing allows us to use the product of LacZ translation, beta-galactosidase (β-gal), as a proxy for cFos and neuronal activation state (Figure 1B). Litters were culled to 8-10 pups on postnatal day (P)1 and remained with the mother until weaning on P21. Between P21 and 25, offspring were genotyped via tissue punches sent to a commercial source (TransnetYX, Cordova, TN). Offspring were group housed (3-4 per cage) with same-sex littermates with the presence of both wildtype (WT) and LacZ positive (LacZ+) rats. Animals were maintained in a climate controlled (20–22 °C) vivarium on a 12 h on/off light cycle (lights on at 0700), and *ad libitum* access to food and water. Experiments were conducted in accordance with the National Institutes of Health guidelines for the care and use of animal subjects, and using protocols approved by Binghamton University Institutional Animal Care and Use Committee. All animals in a cage were exposed to water or ethanol, however only LacZ + rats were used in the present study to assess neuronal activation.

### Intermittent Ethanol Exposure

Experimental subjects (AIE group) were exposed to ethanol (4 g/kg, 25% v/v in tap water) every other day for a total of 11 exposures between P25 and 45 *via* intragastric gavage (see Figure 1A). Exposures were conducted by a trained experimenter with intention to limit any possible stress effect of the gavage procedure. Our lab has previously found this exposure results in blood ethanol concentrations ranging from 200 mg/dl at the start (P25) to 125 mg/dl at the end (P45) (Kim et al., 2019). Control rats (Water group) were exposed to an isovolumetric amount of tap water. All exposures occurred between 1,200 and 1,600 h. Following the final exposure, rats were left undisturbed until P70.

### Modified Social Interaction

As in our previous studies (Varlinskaya et al., 2014; Dannenhoffer et al., 2018; Kim et al., 2019; Varlinskaya et al., 2020), social behavior was assessed using a modified social interaction test (Figure 1A). Experimental subjects (LacZ +) were weighed, marked for identification, and placed into a plexiglass apparatus (46.0 × 30.3 × 30.5 cm) consisting of two equally sized compartments separated by a wall with an aperture allowing for free movement between the compartments. Pine shavings were placed at the bottom of the apparatus, and 6% hydrogen peroxide was used to clean between testing. Testing took place between 1,200 and 1,600 h in the presence of white noise and under dim lighting (10–15 lux). Rats were placed into the testing apparatus alone for a 30 min habituation period prior to introduction of a non-familiar, same-sex social partner that weighed between 15 and 30 grams less than the experimental animal. Social partners were non-manipulated WT rats. Subjects were left together for 60 min with only the first 10 min video recorded for later scoring by a trained researcher blind to experimental condition.

Two measures sensitive to anxiogenic manipulations, social investigation and social preference, were the two indices of social behavior used in the present study. Social investigation was scored as any instance in which the experimental animal sniffed the social partner. A social preference/avoidance coefficient was calculated using the following formula: (crossovers to the partner—crossovers away from the partner) / (total # of crossovers to and away from the partner) x 100.

### Tissue Collection and Enzymatic Histology

At the end of the 60 min social interaction period, experimental rats were injected with sodium pentobarbital and transcardially perfused with 0.1 M phosphate buffered saline (PBS) (200 ml) followed by 4% paraformaldehyde in 0.1 M phosphate buffer (PB) (200 ml). Brains were rapidly extracted and post-fixed in 4% paraformaldehyde for 90 min at 4°C. After postfix, brains were transferred to a 30% sucrose in 0.01 M PBS until saturated, flash-frozen in methyl-butane, and stored at −80°C until processing. In addition to behaviorally tested subjects, a separate cohort of AIE- or water-exposed subjects that did not experience the social context were taken from their home cage environment to obtain control tissue that was processed in a similar fashion. This additional cohort was used to verify whether observed differences in neural activity between water and AIE subjects were due to social interaction or AIE alone.

Using a cryostat (CM 1860, Leica Biosystems, IL, United States), 30 µm coronal slices were collected into an antifreeze solution (30% ethylene glycol, 30% glycerol, 0.05M PBS) and stored at −20 °C. Every 6^th^ non-adjacent section separated by 150 µm of each region of interest was subjected to X-gal enzymatic histology such that at least three bilateral sections per animal were analyzed in accordance with previous literature (Harrison et al., 2008). In short, slices were fixed (0.1 M PB supplemented with 5 mM EGTA) for 15 min followed by two 5-min washes using PB. Slices were then transferred to a staining buffer (0.1 M PB with 2 mM MGCl_2_, 5 mM potassium ferrocyanide, and 5 mM potassium ferricyanide) containing x-gal stock (5-bromo-4-chloro-3-indolyl-β-d-galactoside in dimethylformamide) in a dilution of 1:50 and placed at 37°C overnight. Slices were then washed twice for 10 min each and mounted on charged microscopy slides (Histobond, VWR, PA, United States). Mounted tissue was dehydrated and counterstained using eosin to aid in visualization of β-gal labeling (for representative image see Figure 1C).

### Image Acquisition and Analysis

β-gal stained slices were imaged using an Olympus Research Slide Scanner VS.200 (Olympus, PA, United States) using a 10x magnification objective. Using Olympus ASW software, regions of interest were identified from coronal images and cropped for image analysis. Images were transferred to HALO image analysis platform (Indica Labs, NM, United States) and β-gal containing cells were automatically identified based expression of indigo blue staining and on soma size and roundness. The automated identification of β-gal positive (β-gal+) cells was verified using hand-counted images with over 95% reliability between observation methods.

### Regions of Interest

Regions of interest (ROIs) were selected based on the reported contribution of these regions to regulation of social behavior (Newman, 1999; Yizhar, 2012; Allsop et al., 2014; Ko, 2017). We evaluated β-gal expression in 11 ROIs; PrL, IL, vOFC, lOFC, NAcC, NAcSh, LS, dBNST, AH, CeA, and BLA. Coordinates for ROIs can be found in Table 1. Within each region, a 400 μm^2^ area was extracted, and the number of β-gal + cells were counted and converted to a number/mm^2^.

**TABLE 1:** Anatomic location of each region of interest.

Region of interest	Location (in References to Bregma)
Lateral Orbitofrontal (lOFC)	4.68–2.76 mm
Ventral Orbitofrontal (vOFC)	4.68–3.00 mm
Prelimbic cortex (PrL)	4.40–2.52 mm
Infralimbic cortex (IL)	3.72–2.52 mm
Nucleus Accumbens Core (NAcC)	2.76–1.08 mm
Nucleus Accumbens Shell (NAcSh)	2.76–0.96 mm
Lateral Septum (LS)	2.16–0.00 mm
Bed Nucleus of the Stria Terminalis (BNST)	0.24 to −0.60 mm
Anterior Hypothalamus (AH)	−1.56 to −2.16 mm
Central Amygdala (CeA)	−1.56 to −3.24 mm
Basolateral Amygdala (BLA)	−1.72 to −3.36 mm

### Statistical Approach and Analysis

We have previously found AIE-induced sex-specific alterations to social behavior (Varlinskaya et al., 2014; Dannenhoffer et al., 2018; Varlinskaya et al., 2020) therefore all analyses were restricted to within and not between each sex separately. Independent samples t-tests were used to compare social investigation and social preference of water- and AIE-exposed rats within each sex. To explore the relationship of social behavior and neuronal activation (indexed via β-gal + cell count) within a brain region, linear regressions were conducted separately for each adolescent exposure condition in males and females. Correlation analyses were then used to determine the directionality and strength of the relationships. In order to determine whether AIE affected β-gal expression in response to a social stimulus within each ROI, we initially compared the number of β-gal + cells in water- and AIE-exposed socially tested subjects using independent samples t-tests separately in males and females. Once ROIs with significant differences between the two adolescent exposure conditions were identified, we further assessed whether these differences were in fact related to social testing and not an effect of AIE per se. To do so, we used a 2 testing context (home cage, social testing) x 2 adolescent exposure (water, AIE) analyses of variance (ANOVAs). This approach allowed us to identify AIE-associated changes in β-gal expression associated with responding to the social stimulus. Significant main effects and/or interactions were further assessed using Bonferroni’s multiple comparisons tests.

Outliers were identified using values outside 2 standard deviations. One water male, one water female, and one AIE female were removed due to being outliers in behavior and were not included in analyses in β-gal expression. In addition, a low number of outliers were identified within each ROI with final group sizes between 10 - 12.

## Results

### Social Preference and Investigation

**FIGURE 2:**
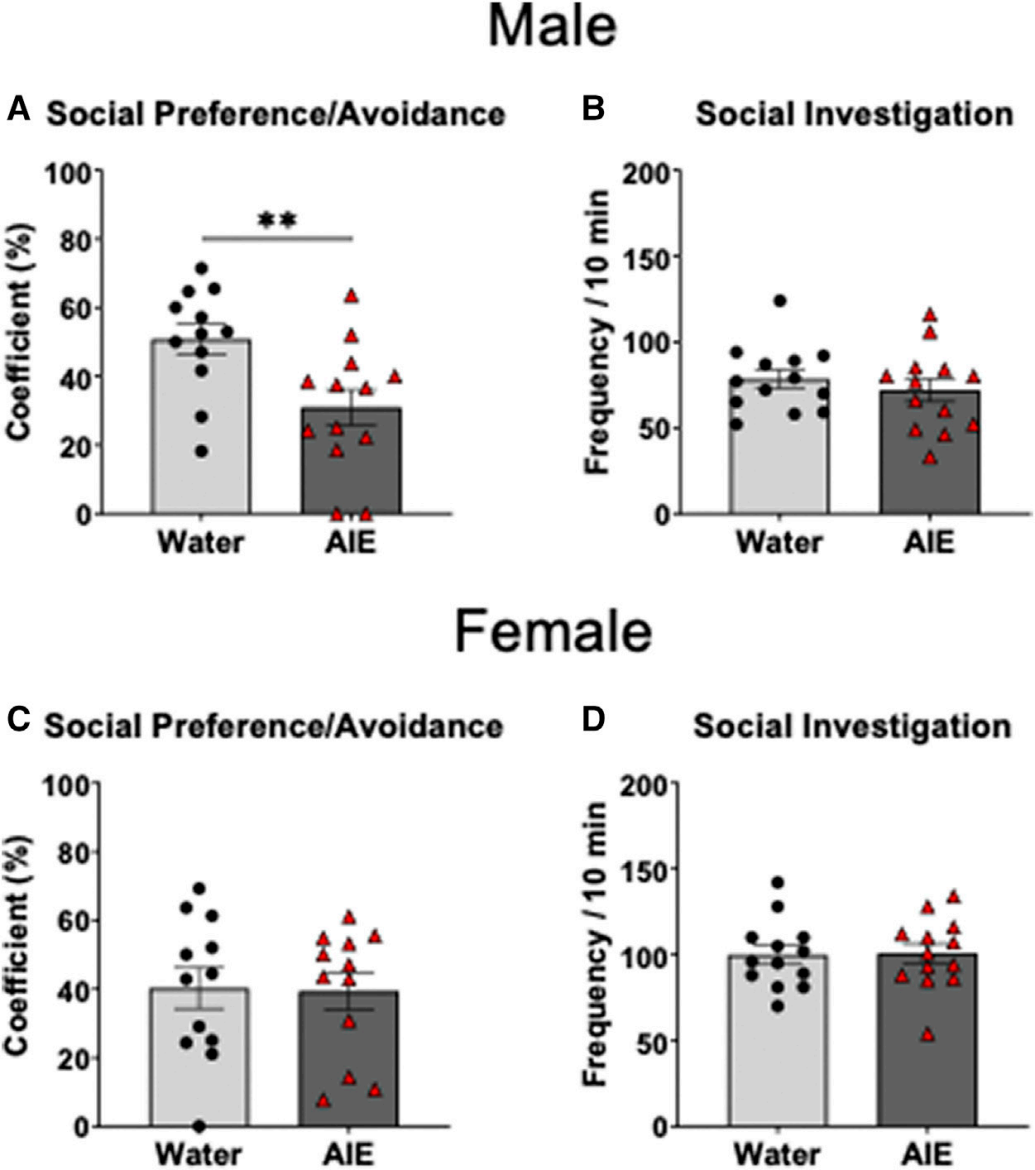
Effects of adolescent intermittent ethanol (AIE) exposure on social preference/avoidance and social investigation in adult male **(A–B)** and female **(C–D)** cFos-LacZ rats. Social investigation was not altered by AIE, while social preference was decreased in AIE-exposed males relative to water-exposed controls, with no AIE effect evident in females. Significant differences between adolescent exposure conditions within each sex are marked with ^**^ *p* < 0.01.

Independent samples *t*-test of the social preference/avoidance coefficient revealed that social preference of AIE-exposed males was significantly lower than in their water-exposed counterparts (t _23_ = 2.90, *p* < 0.01, Figure 2A). As can be seen in Figure 2C, there was no difference in social preference between females exposed to water and AIE (*p* > 0.05). However, the independent samples t-tests of social investigation showed no significant difference between adolescent exposure conditions for both males and females (*p* > 0.05, Figures 2B,D respectively).

### Relationship Between Social Preference and Neuronal Activation

Linear regressions were performed for social interaction and social preference within each adolescent exposure condition and sex. In water-exposed males, social preference predicted β-gal + cell count in the dBNST (*r* ^*2*^ = 0.37, *F*_(1,9)_ = 5.22, *p* < 0.05) with a strong positive relationship between these variables, *r* = 0.61. For AIE-exposed males, linear regressions identified significant relationships between social preference and β-gal + cell count in the dBNST *r* ^*2*^ = 0.34, *F*_(1,10)_ = 5.19, *p* < 0.05, LS, *r* ^*2*^ = 0.35, *F*_(1,10)_ = 5.32, *p* < 0.05, and vOFC, *r* ^*2*^ = 0.44, *F*_(1,11)_ = 8.66, *p* < 0.05. Correlation analyses revealed strong negative relationships between social preference and β-gal expression in all three regions (dBNST, *r* = −0.58, LS, *r* = −0.59, vOFC, *r* = −0.66, see Table 2). No other significant relationships were identified in males (see Table 2).

**TABLE 2:** The relationship between social preference/avoidance coefficient and the number of β-gal positive cells for each region of interest of water and AIE exposed males and females assessed with Pearson correlation and linear regression analyses.

Region of interest	Male						Female					
	Water			AIE			Water			AIE		
	*r*	*r* ^*2*^	*p*	*r*	*r* ^*2*^	*p*	*r*	*r* ^*2*^	*p*	*r*	*r* ^*2*^	*p*
PrL	−0.26	0.07	0.41	−0.23	0.05	0.47	−0.13	0.02	0.68	−0.34	0.11	0.29
IL	−0.14	0.02	0.66	−0.15	0.02	0.62	0.14	0.02	0.69	−0.30	0.09	0.36
vOFC	−0.16	0.02	0.64	−**0.66**	**0.44**	**0.01**	0.26	0.07	0.45	−0.52	0.27	0.10
lOFC	−0.09	0.01	0.77	−0.47	0.21	0.11	0.25	0.06	0.44	−0.38	0.14	0.22
NAcC	0.24	0.06	0.45	−0.29	0.08	0.36	−0.03	0.00	0.92	−**0.60**	**0.37**	**0.03**
NAcSh	0.31	0.10	0.33	−0.13	0.02	0.71	−0.13	0.02	0.70	−**0.65**	**0.43**	**0.02**
LS	−0.26	0.07	0.41	−**0.59**	**0.35**	**0.04**	−0.18	0.03	0.58	−0.34	0.12	0.27
BNST	**0.61**	**0.37**	**0.05**	−**0.58**	**0.34**	**0.05**	0.02	0.00	0.95	−0.17	0.03	0.60
AH	0.01	0.00	0.99	−0.17	0.03	0.60	−0.30	0.09	0.35	−0.03	0.00	0.92
CeA	0.15	0.02	0.67	−0.25	0.06	0.42	−0.05	0.00	0.87	0.11	0.01	0.72
BLA	−0.22	0.05	0.51	−0.39	0.15	0.24	0.34	0.11	0.31	−0.21	0.04	0.52

Bold text represents significant correlations and linear regressions.

For females, linear regression analyses revealed no significant relationships between social preference and β-gal + cells in the water-exposed females across any of the ROIs analyzed. In AIE-exposed females, regression analyses found relationships between social preference and β-gal expression in both the NAcC (*r* ^*2*^ = 0.38, *F*_(1,10)_ = 5.76, *p* < 0.05) and NAcSh (*r* ^*2*^ = 0.43, *F*_(1,10)_ = 7.44, *p* < 0.05), with each being negatively correlated to social preference (NacC, *r* = −0.60 and NAcSh, *r =* −0.65). No other relationships were evident across the other ROIs (Table 2).

### Relationship Between Social Investigation and Neuronal Activation

For social investigation, linear regression analyses revealed relationships between social investigation and β-gal + neurons for water-exposed males in the PrL (*r* ^*2*^ = 0.37, *F*_(1, 10)_ = 5.76, *p* < 0.05), IL (*r* ^*2*^ = 0.49, *F*_(1,10)_ = 9.62, *p* = 0.01), and NAcC (*r* ^*2*^ = 0.45, *F*_(1,10)_ = 8.22, *p* < 0.05). All three relationships in water-exposed males was positively correlated (PrL *r* = 0.61, IL *r* = 0.70, NAcC *r* = 0.67). For water exposed females, linear regressions revealed a relationship only between investigation and β-gal expression in the vOFC (*r* ^*2*^ = 0.50, *F*_(1,9)_ = 9.18, *p* = 0.01), with a strong negative correlation between these variables (*r* = -0.71). In AIE-exposed males and females, no significant linear regressions or correlations between social investigation and β-gal expression in any of the ROI analyzed were identified (see Table 3).

**TABLE 3:** The relationship between social investigation and the number of β-gal positive cells for each region of interest of water and AIE exposed males and females assessed with Pearson correlation and linear regression analyses.

Region of interest	Male						Female					
	Water			AIE			Water			AIE		
	*r*	*r* ^*2*^	*p*	*r*	*r* ^*2*^	*p*	*r*	*r* ^*2*^	*p*	*r*	*r* ^*2*^	*p*
PrL	**0.61**	**0.37**	**0.04**	−0.26	0.07	0.42	−0.51	0.26	0.09	−0.27	0.07	0.39
IL	**0.70**	**0.49**	**0.01**	−0.18	0.03	0.55	−0.26	0.07	0.43	0.09	0.01	0.78
vOFC	0.57	0.32	0.06	−0.21	0.05	0.48	−**0.71**	**0.50**	**0.01**	−0.21	0.05	0.53
lOFC	0.51	0.26	0.09	−0.00	0.00	1.00	−0.35	0.12	0.26	0.19	0.03	0.56
NAcC	**0.67**	**0.45**	**0.02**	0.37	0.14	0.24	−0.55	0.31	0.06	0.12	0.01	0.71
NAcSh	0.50	0.25	0.10	0.24	0.06	0.48	−0.33	0.11	0.30	−0.14	0.02	0.67
LS	0.17	0.03	0.59	0.11	0.01	0.75	−0.25	0.06	0.43	−0.30	0.09	0.34
BNST	−0.09	0.01	0.78	0.04	0.00	0.89	−0.55	0.30	0.07	−0.16	0.03	0.62
AH	0.45	0.20	0.16	0.07	0.01	0.82	0.29	0.08	0.36	−0.30	0.09	0.34
CeA	0.36	0.13	0.28	−0.13	0.02	0.69	0.17	0.03	0.60	0.34	0.11	0.28
BLA	0.52	0.27	0.10	0.06	0.00	0.87	−0.05	0.00	0.88	−0.17	0.03	0.60

Bold text represents significant correlations and linear regressions.

### Neuronal Activation Following Social Stimulus and Home Cage Conditions

To determine ROIs that were different between exposure conditions following social testing, we conducted independent samples t-tests for each ROI within each sex (see Table 4). In males, only the CeA differed, whereas in females both subregions of the OFC (vOFC and lOFC) were affected by AIE. No other ROI for either sex was identified as having differential β-gal labeling between conditions following social interaction (Table 4).

**TABLE 4:** Independent samples t-test results for social stimulus associated β-gal labeling between adolescent exposure conditions (water; AIE) within each region of interest.

Region of interest	Results	
	Male	Female
PrL	*t*_22_ = 1.31, *p* = 0.20	*t*_22_ = 0.29, *p* = 0.78
IL	*t*_23_ = 0.04, *p* = 0.97	*t*_20_ = 0.72, *p* = 0.48
vOFC	*t*_23_ = 0.27, *p* = 0.79	***t***_20_ **= 2.05, *p* = 0.05**
lOFC	*t*_21_ = 1.60, *p* = 0.12	***t***_22_ **= 2.21, *p* = 0.03**
NAcC	*t*_22_ = 0.27, *p* = 0.79	*t*_22_ = 0.20, *p* = 0.84
NAcSh	*t*_21_ = 0.82, *p* = 0.42	*t*_22_ = 0.17, *p* = 0.86
LS	*t*_22_ = 0.29, *p* = 0.77	*t*_22_ = 0.19, *p* = 0.85
BNST	*t*_21_ = 0.80, *p* = 0.43	*t*_22_ = 0.24, *p* = 0.81
AH	*t*_21_ = 0.48, *p* = 0.63	*t*_22_ = 0.38, *p* = 0.71
CeA	***t***_21_ **= 2.73, *p* = 0.01**	*t*_22_ = 0.57, *p* = 0.58
BLA	*t*_20_ = 0.56, *p* = 0.58	*t*_21_ = 1.78, *p* = 0.09

Bold text represents significant differences.

**FIGURE 3:**
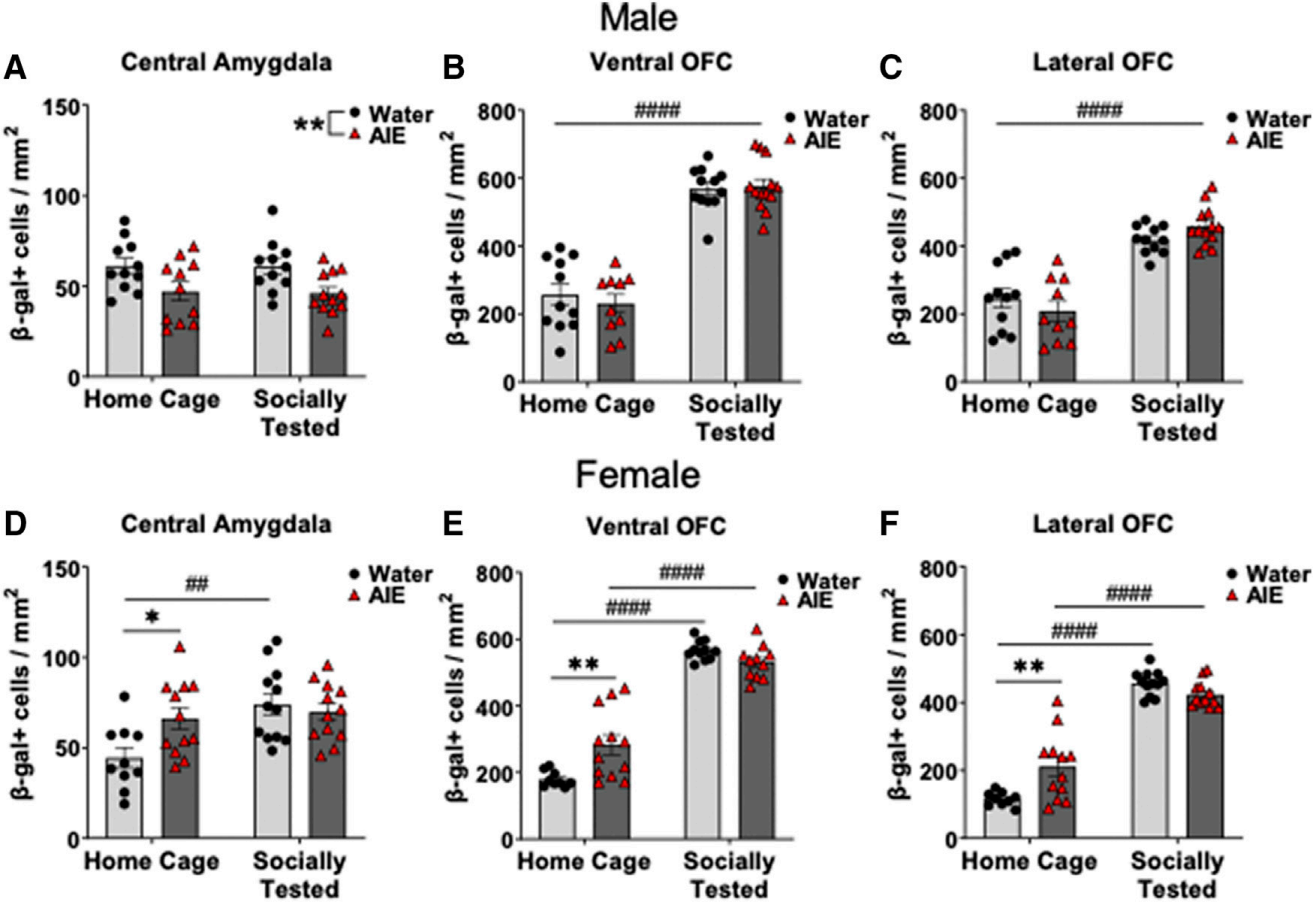
Effects of adolescent intermittent ethanol (AIE) exposure on neuronal activation of **(A,D)** the central amygdala (CeA), **(B,E)** ventral orbitofrontal cortex (vOFC), and **(C,F)** lateral orbitofrontal cortex (lOFC) in home cage and socially tested adult males and females. Number of β-gal positive cells in the CeA was significantly lower in AIE-exposed males than in water-exposed controls, with no difference between social testing conditions. In females, CeA β-gal expression was greater for AIE-exposed home cage subjects compared water-exposed controls. Socially tested, water-exposed females had increased CeA β-gal expression compared to home cage water-exposed females. β-gal + cells were greater in both subregions of the OFC in socially tested males, regardless of adolescent exposure. In females, the number of β-gal + cells in the vOFC and lOFC was higher in home cage AIE-exposed females than in their water-exposed counterparts. Additionally, social testing in females led to increases β-gal expression in both OFC subregions, regardless of adolescent exposure. Asterisks denote significant differences between adolescent exposure conditions, ^*^ *p* ≤ 0.05, ^**^ *p* = 0.01 whereas ^#^ denote significant differences between testing conditions, ^##^ *p* < 0.01, ^####^ *p* < 0.0001.

To assess whether our significant findings in β-gal labeling were the result of a social stimulus, we compared home cage subjects and socially tested subjects using two-way ANOVAs. In male CeA β-gal expression, analysis revealed a main effect of adolescent exposure condition (*F*_(1,41)_ = 11.31, *p* < 0.01), but not for behavioral testing condition or interaction. As seen in Figure 3A, AIE-exposed males had lower β-gal expression in the CeA in comparison to water-exposed counterparts, regardless of testing condition. Analysis of male OFC β-gal expression revealed a main effect of testing condition in both the vOFC (*F*_(1,42)_ = 178.00, *p* < 0.0001) and lOFC (*F*_(1,41)_ = 89.90, *p* < 0.0001). Regardless of adolescent exposure condition, socially tested males had greater β-gal labeling in the OFC compared to home-cage subjects (Figures 3B,C).

In females, analysis of CeA β-gal + cells revealed a main effect of testing context (*F*_(1,42)_ = 8.73, *p* < 0.01), as well as a testing context by adolescent exposure interaction (*F*_(1,42)_ = 5.40, *p* < 0.05). Follow up comparisons of this interaction revealed a difference between adolescent exposure conditions. In home cage controls, AIE-exposed females had more β-gal + cells than water-exposed females (*p* < 0.05, Figure 3D), whereas socially tested water-exposed females having more β-gal expression than home-cage control females (*p* < 0.01, Figure 3D).

For the vOFC in females, analysis revealed a main effect of testing context (*F*_(1,39)_ = 269.90, *p* < 0.0001), and a testing context by adolescent exposure interaction (*F*_(1,39)_ = 12.77, *p* = 0.001). Follow up comparisons identified a significant difference between water- and AIE-exposed females in the home cage condition (*p* < 0.01), with AIE-exposed females having more β-gal + neurons than their water-exposed counterparts (see Figure 3E). In addition, differences were found between home cage and socially tested subjects’ conditions in both exposure conditions (*p* < 0.0001), with greater expression of β-gal in socially tested females than in home cage controls (Figure 3E).

Finally, analysis of β-gal labeling in the lOFC also revealed a main effect of testing context (*F*_(1,41)_ = 249.10, *p* < 0.0001), and a testing context by adolescent exposure interaction, (*F*_(1,41)_ = 13.60, *p* < 0.001). Similar to the vOFC, follow up comparisons in the lOFC revealed a difference between adolescent exposures in the home cage condition (*p* = 0.001), with AIE-exposed females having a greater number of β-gal + cells than their water-exposed counterparts. Socially tested females in both exposure conditions also had greater β-gal expression relative to home cage subjects (*p* < 0.0001, see Figure 3F).

## Discussion

In this study, we aimed to determine brain regions involved in modulation of social behavior that might contribute to AIE-induced social alterations. Similar to our previous findings in non-transgenic Sprague Dawley rats (Varlinskaya et al., 2014; Varlinskaya et al., 2017; Dannenhoffer et al., 2018; Varlinskaya et al., 2020), AIE led to a sex-specific social impairment in transgenic cFos-LacZ animals, with only males displaying a decreased social preference following adolescent ethanol exposure.

The analyses of relationships between social behaviors and neuronal activation of the ROIs revealed that these relationships are altered by AIE, with these alterations evident in both males and females. Specifically, in water-exposed control males, frequency of social investigation was found to be positively correlated with the number of β-gal + neurons in the PrL, IL, and NAcC, suggesting an involvement of these brain regions in regulation of male social investigation. In contrast, no relationship between activation of these regions and social investigation was evident in AIE-exposed males, suggesting AIE-induced alterations of the neural circuitry involved in modulation of social investigation. Alterations of the mPFC and NAc induced by AIE exposure may limit the ability of these regions to contribute to social investigation. Identified AIE-induced changes in mPFC inhibitory signaling include reduced interneuron intrinsic excitability (Trantham-Davidson et al., 2017), increased inhibitory signaling onto layer V pyramidal neurons (Centanni et al., 2017), and decreases in the number of parvalbumin expressing interneurons (Rice et al., 2019), with these changes to inhibitory signaling in this region potentially contributing to a dysregulated activation associated with social investigation. Additionally, AIE has been found to blunt stimulus evoked dopamine signaling in the NAc (Shnitko et al., 2016) and potentiate kappa opioid receptor function in mediating dopamine release (Spodnick et al., 2020). All these alterations might also contribute to male-specific AIE-associated changes observed in the present study.

Activation of mPFC and NAcC of females did not contribute to social investigation in both adolescent exposure conditions, suggesting sex-specific involvement of these regions in social investigation and AIE-induced deficits. In contrast to males, social investigation in water-exposed females was negatively correlated with vOFC neuronal activation, with no such relationship evident in AIE females, suggesting AIE-associated neuronal alterations in modulation of social behavior in females. As mentioned above, the OFC modulates various social behaviors in males, however the involvement of this region in female social investigation has not been thoroughly studied. It was surprising to find a negative relationship between OFC activity and female social investigation in the current study, given that OFC lesions reduced social play (Van Kerkhof et al., 2013) and increased aggression (Rudebeck et al., 2007). However, the involvement of the OFC in social behaviors has not been evaluated in females, therefore it is likely that regulation of social investigation by the OFC differs between females and males. Sex differences in the OFC are also evident, with females having more myelin basic protein (Bayless and Daniel, 2015; Darling and Daniel, 2019) and greater OFC volume than males (Goldstein et al., 2001; Gur et al., 2002), although the contribution of these sex-differences to social behavior remain unknown.

The relationship between social preference and neuronal activation differed from that of social investigation. In water-exposed control males, β-gal expression in the dBNST was positively correlated with social preference, while AIE reversed this relationship, suggesting AIE-associated alterations in the role of the dBNST in social preference. Indeed, the BNST has been shown to assess social contexts in order to select an appropriate behavioral response, while alterations of socially relevant BNST systems by different insults result in social dysfunctions (Flanigan and Kash, 2020). In addition, social preference was negatively correlated with activation of the vOFC and LS in AIE-exposed males, whereas it was negatively correlated with activation of the NAcC and NAcSh in AIE-exposed females. These findings suggest that AIE produces sex- and region-specific dysfunctions in brain regions implicated in social preference/avoidance. In other words, activation of brain regions that are normally implicated in social behavior (Vanderschuren et al., 2016; Menon et al., 2021) is associated with lower social preference and/or social avoidance following AIE, potentially due to AIE-induced changes in either expression of certain cell types expressing β-gal or alterations to connectivity, and/or neurotransmitter release within these regions compared to control animals. Clearly, more work is needed to understand neural mechanisms of dysfunctions in brain regions implicated in social preference/avoidance sex-specifically affected by AIE. Furthermore, the results of the present study suggest that social investigation and social preference of males have different underlying circuitry, with IL, PrL, and NAcC implicated in regulation of social investigation and BNST contributing to social preference. This finding was not surprising, given that social investigation is a form of exploratory behavior that provides needed information about a novel conspecific, whereas the coefficient of social preference/avoidance reflects approach/avoidance behavior under social circumstances.

When assessing neuronal activation following an interaction with a social partner and under home cage condition in cFos-LacZ rats using β-gal as the proxy for cFos, we found that AIE and water-exposed males had differential activation of the CeA regardless of testing condition, with AIE-exposed males having less β-gal + cells in the CeA than water-exposed controls. It is likely that in males, AIE produces a long-lasting reduction in CeA activity indexed through β-gal expression. This finding is in accordance with others that have shown AIE-associated alterations in the CeA, including decreases expression the activity-regulated cytoskeletal (Arc) gene (Pandey et al., 2015; Kyzar et al., 2019), another early gene marker used to assess neuronal activation (Korb and Finkbeiner, 2011). We cannot preclude, however, that the attenuated activation state of the CeA in AIE males plays a role in AIE-induced social impairments by predisposing these males to avoidance behavior. Future studies are needed to further assess the role of the CeA in social deficits associated with AIE. Although no difference in CeA neuronal activation between exposure conditions was evident in socially tested females, we found that β-gal expression in the CeA under home cage conditions was also affected by AIE. However, in contrast to males in this study and others (Pandey et al., 2015; Kyzar et al., 2019), AIE-exposed females had more β-gal + cells in the CeA than their water-exposed counterparts. Interestingly, social behavior led to a recruitment of neurons in the CeA of control females, however this effect was absent in AIE-exposed females. Although no behavioral difference was observed between female exposure conditions, it is possible that social behavior in each of these exposure groups relies on differential activation of brain regions such as the CeA. These findings add to the growing literature suggesting that AIE leads to sex-specific alterations (Robinson et al., 2021), including opposite effects of AIE on CeA in males and females.

In females, social investigation and social preference did not differ as a function of adolescent exposure, however, the OFC was differentially activated in water- and AIE-exposed females. In, ventral and lateral subregions of the OFC, AIE-exposed females had lower β-gal expression than their water-exposed counterparts, with these differences evident in both home cage controls and socially tested subject, indicating AIE-associated alterations of the OFC in females. Alterations of the OFC following AIE have been recently identified. For example, AIE leads to reduced cholinergic markers and lower acetylcholine release in the OFC during a spontaneous alternation task, although these results were similar between sexes (Kipp et al., 2021). AIE also results in increased neuroimmune signaling in the OFC of males (Vetreno et al., 2013), however this was not assessed in females. Furthermore, AIE has been shown to increase perineuronal net (PNNs) expression in the OFC (Coleman et al., 2014) and given that these nets mediate inhibitory signaling (Bosiacki et al., 2019; Carceller et al., 2020), it is possible that PNN upregulation contributes to the increased neuronal activity among AIE-exposed females evident in the current study. The increases in neuroinflammation and perineuronal nets following AIE have only been studied in males, and more work is needed to determine whether these changes are evident in females and whether they contribute to differences in basal activity of the OFC. In both sexes, however, exposure to a social stimulus enhanced neuronal activation of the vOFC and lOFC, with greater number of β-gal-containing neurons evident in socially tested animals than home cage controls regardless of adolescent exposure condition. This finding suggest that the OFC contributes to social responding of adult males and females, and that the OFC is still responsive to social stimuli after AIE. Evidence supports a role of the OFC in modulating different social behaviors, including social play (Van Kerkhof et al., 2013), aggression (Rudebeck et al., 2007), and dominance (Kolb et al., 2004), whereas no information is available regarding possible involvement of the OFC in regulation of social investigation and/or social preference in males and females.

The present study has certain limitations. Our initial analyses using independent samples t-tests revealed significant differences between socially tested adolescent exposure conditions in the vOFC and lOFC however when using two-way ANOVAs these comparisons failed to reach significance. It is likely that our effect was small in these analyses and our power was too low for identification of these differences with additional analyses. Future work will determine whether these differences in OFC activation between socially tested females can be replicated. Moreover, a lack of differences in activation of a certain brain region does not imply that the same types of neurons were activated by a social stimulus in water-exposed controls and AIE animals. Therefore, our future studies will focus on identification of subtypes of neurons activated by exposure to a social partner in ROIs activation of which was correlated either with social preference or social investigation. In addition, the use of a single immediate early gene limits our understanding of the timeframe in which a neuron was active. The use of either different immediate early genes or different timepoints may be appropriate to further assess the activation of the ROIs in relation to social behavior. Furthermore, we cannot rule out that changes associated with social interaction relative to home cage levels of β-gal expression can be detected in other ROIs examined, since baseline neuronal activation was only assessed in regions with significant differences in activation between socially tested water- and AIE-exposed males and females. Future studies will determine whether AIE induces alterations in basal β-gal expression and whether exposure to a social stimulus produces changes relative to baseline condition differentially in AIE animals and water-exposed controls.

Overall, in males, AIE reduced social preference and affected the relationship between social behaviors’ and neuronal activation of IL, PrL, vOFC, NAcC, BNST, and LS. Importantly, although no behavioral alterations were observed in AIE-exposed females, our findings suggest that the relationship between social behaviors and neuronal activation of vOFC, nAcC, and NAcSh were affected by AIE. Further studies need to be conducted to determine neuronal phenotypes are activated in response to a social stimulus and to test whether these phenotypes differ as a result of AIE exposure in males and females.

## Data Availability

The raw data supporting the conclusion of this article will be made available by the authors, without undue reservation.
